# Supplementary figures and images for: Ferulic acid attenuates high glucose-induced apoptosis in retinal pigment epithelium cells and protects retina in db/db mice
Source: PeerJ. 2022 May 31;10:e13375. doi: 10.7717/peerj.13375 (PMC9165606; doi:10.7717/peerj.13375)

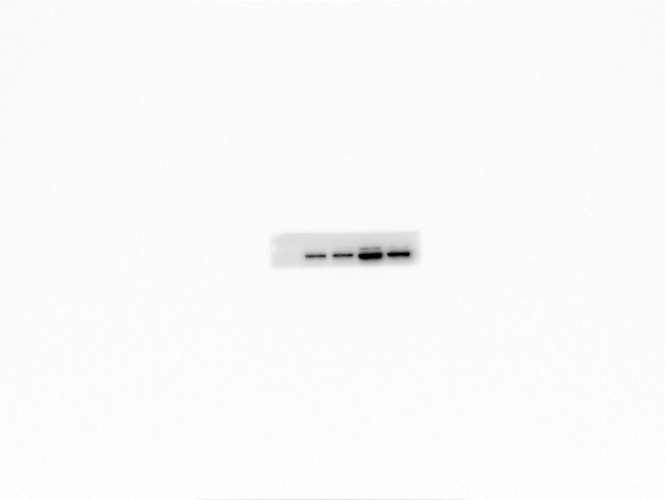

Supplement: Supplemental Information 1 [file peerj-10-13375-s001.zip › Original data/Original data-Figure 2/Figure 2D-Western Blot/BAX.jpg]

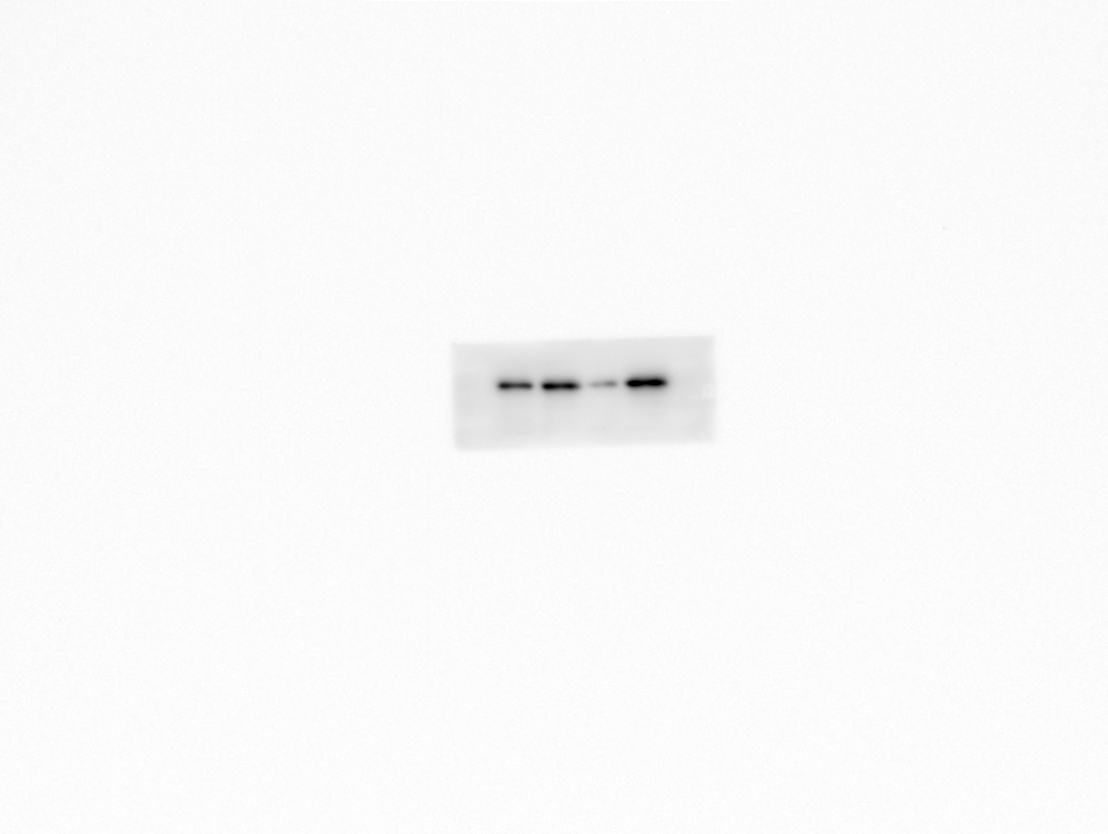

Supplement: Supplemental Information 1 [file peerj-10-13375-s001.zip › Original data/Original data-Figure 2/Figure 2D-Western Blot/Bcl2.jpg]

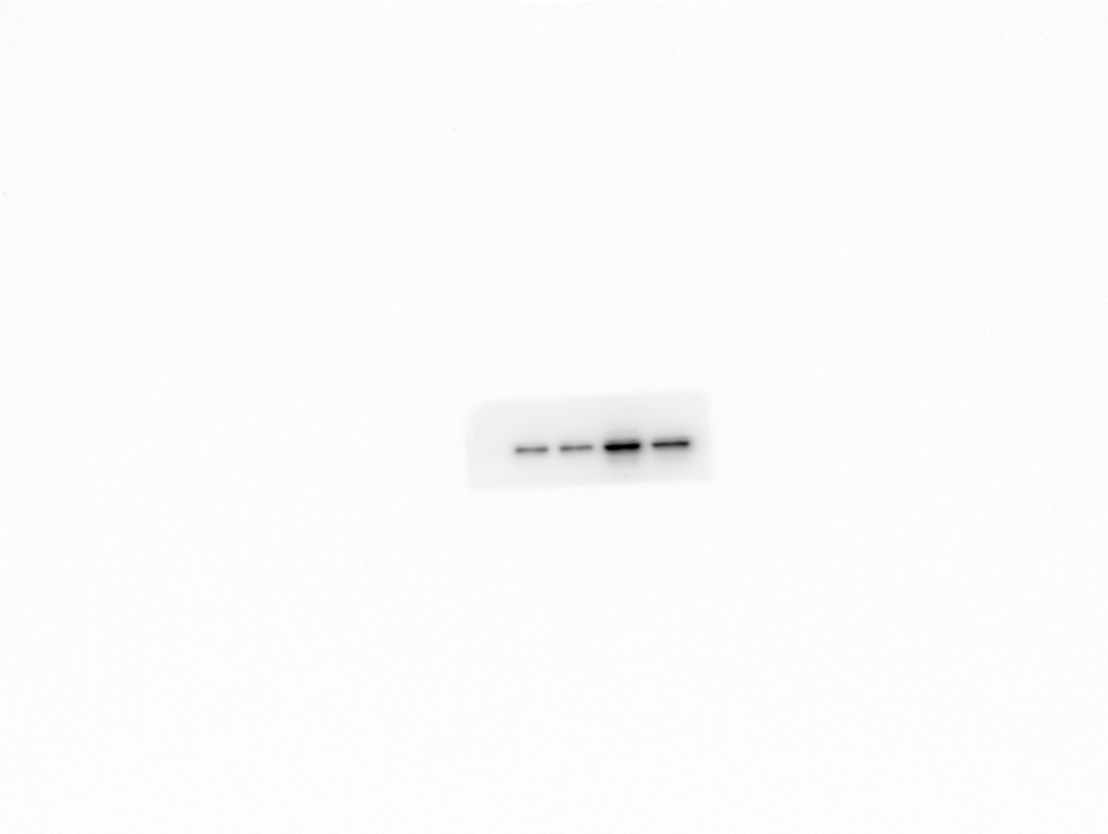

Supplement: Supplemental Information 1 [file peerj-10-13375-s001.zip › Original data/Original data-Figure 2/Figure 2D-Western Blot/Cleaved Caspase3.jpg]

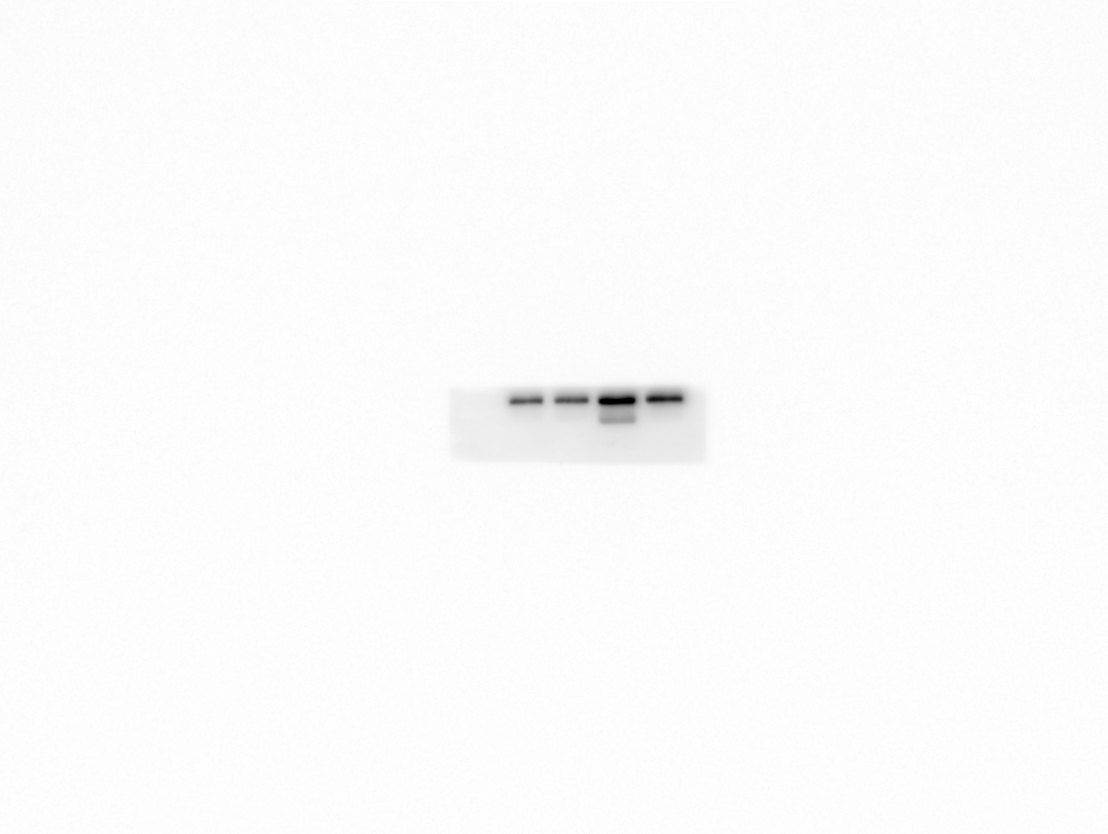

Supplement: Supplemental Information 1 [file peerj-10-13375-s001.zip › Original data/Original data-Figure 2/Figure 2D-Western Blot/P53.jpg]

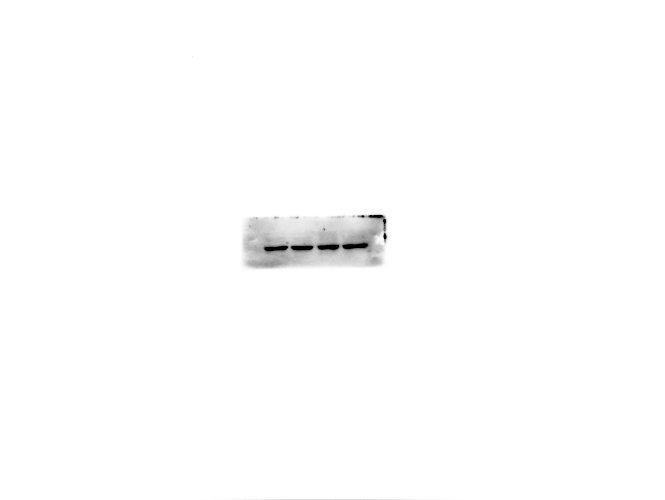

Supplement: Supplemental Information 1 [file peerj-10-13375-s001.zip › Original data/Original data-Figure 2/Figure 2D-Western Blot/a┬-actin.jpg]

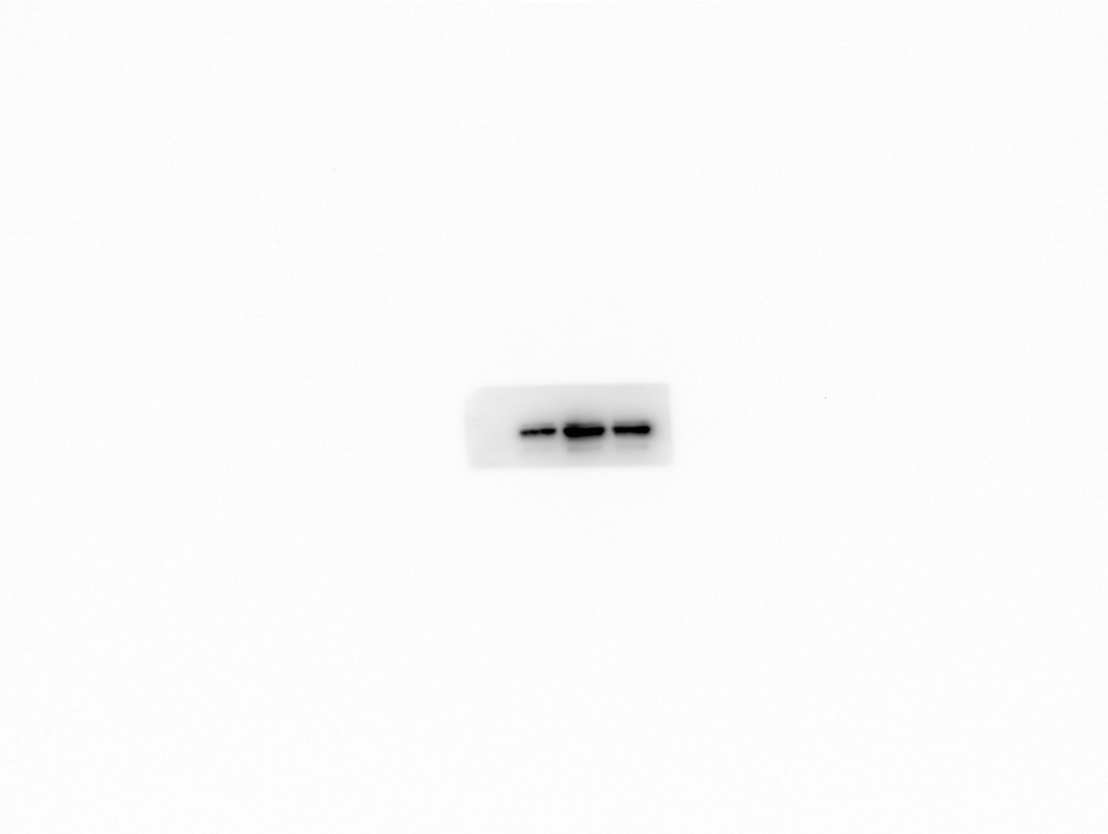

Supplement: Supplemental Information 1 [file peerj-10-13375-s001.zip › Original data/Original data-Figure 6/Figure 6N-Western Blot/BAX.jpg]

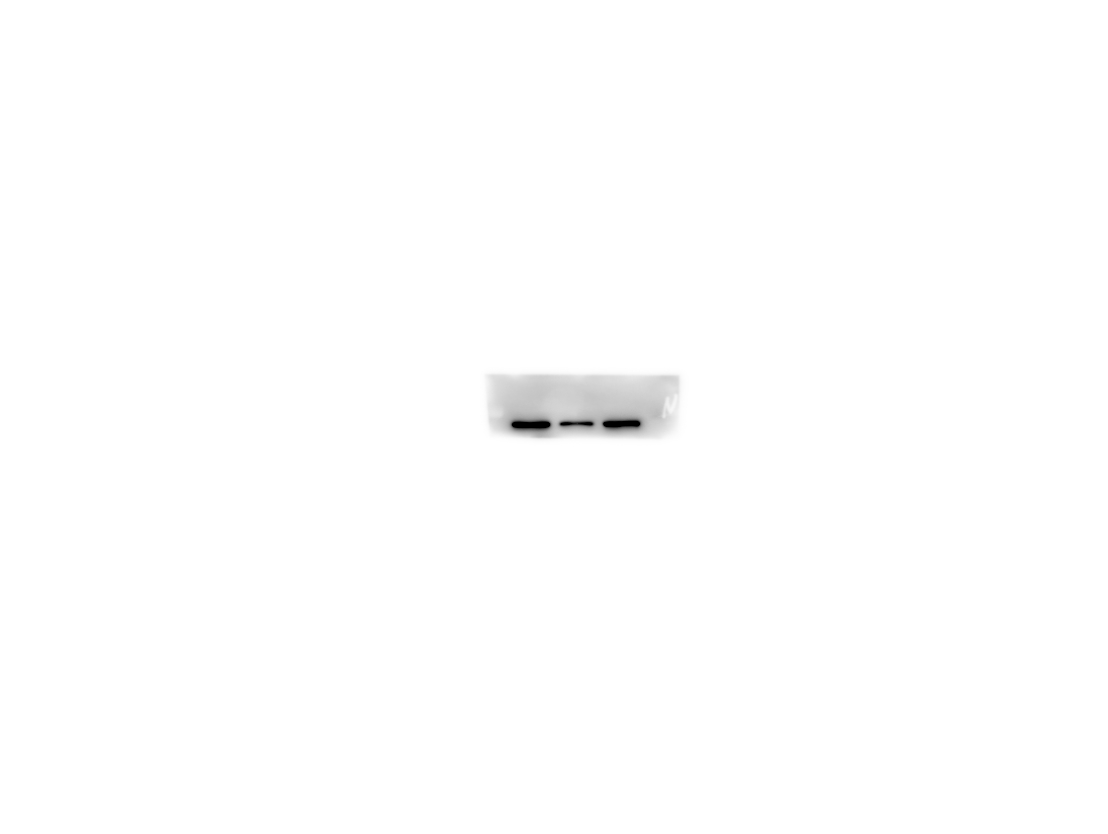

Supplement: Supplemental Information 1 [file peerj-10-13375-s001.zip › Original data/Original data-Figure 6/Figure 6N-Western Blot/Bcl2.jpg]

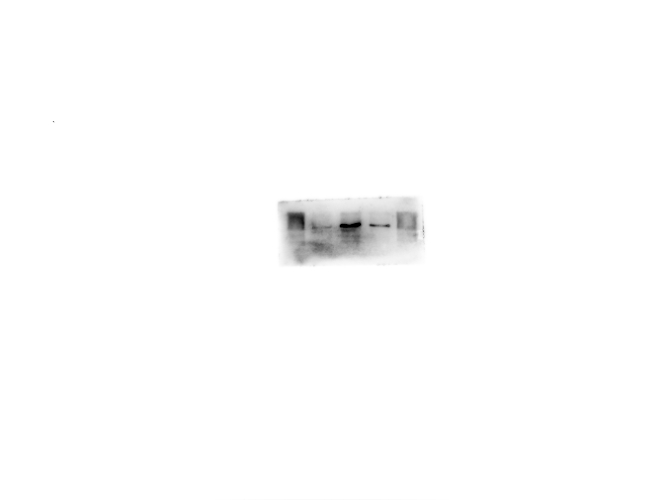

Supplement: Supplemental Information 1 [file peerj-10-13375-s001.zip › Original data/Original data-Figure 6/Figure 6N-Western Blot/Cleaved Caspase3.jpg]

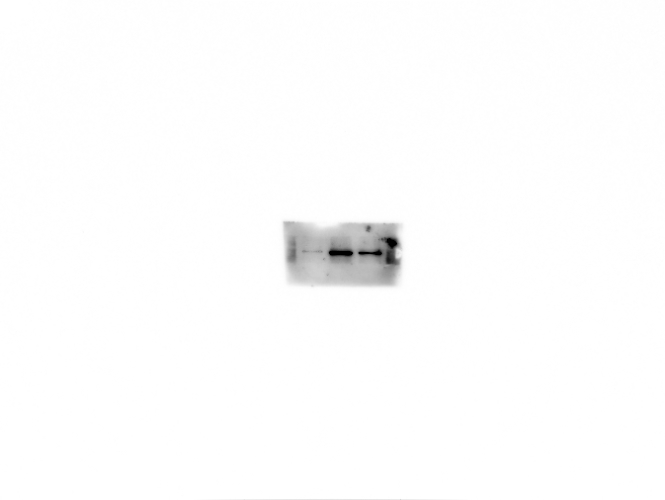

Supplement: Supplemental Information 1 [file peerj-10-13375-s001.zip › Original data/Original data-Figure 6/Figure 6N-Western Blot/P53.jpg]

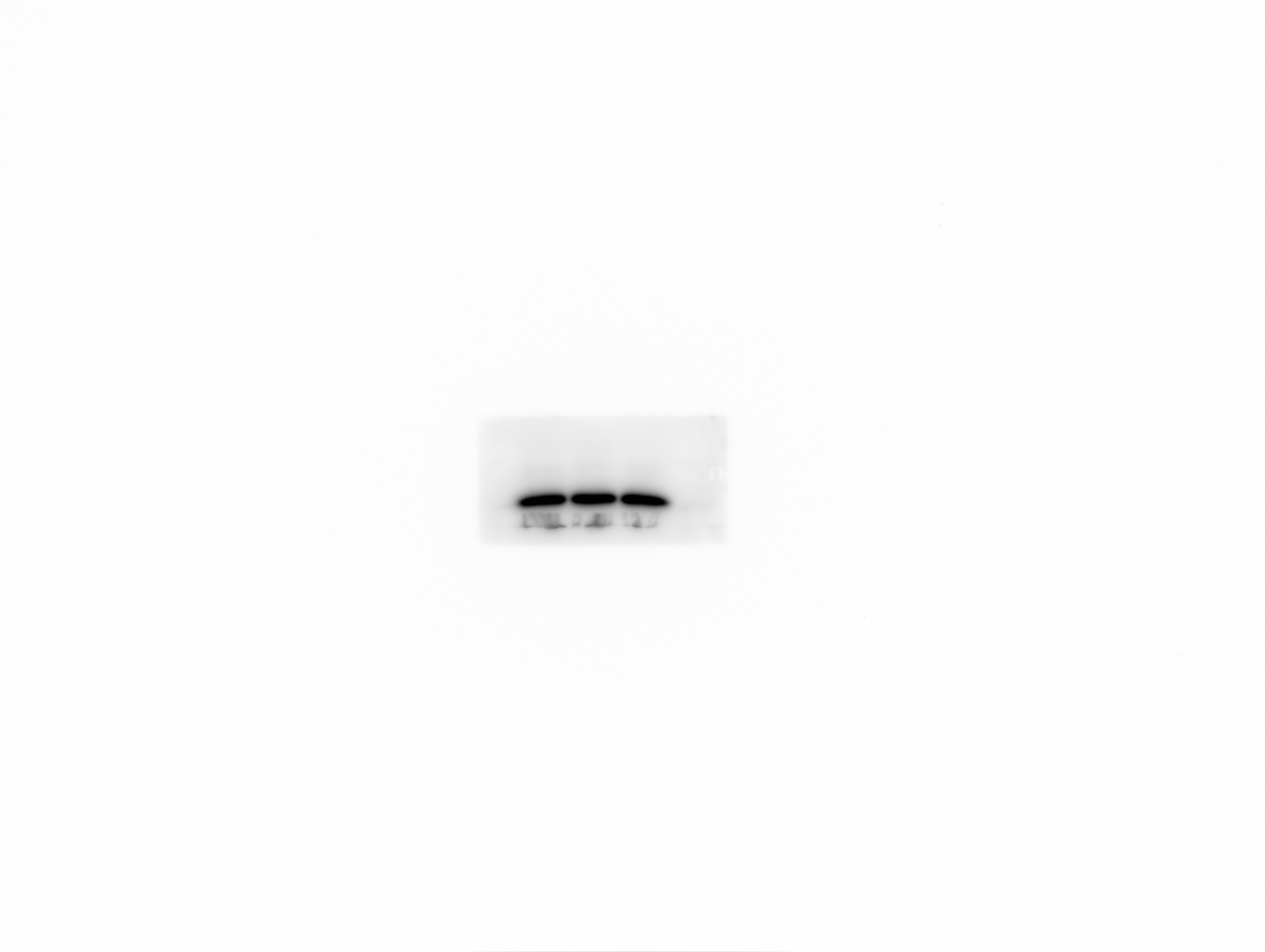

Supplement: Supplemental Information 1 [file peerj-10-13375-s001.zip › Original data/Original data-Figure 6/Figure 6N-Western Blot/a┬-actin.jpg]
